# Effect of a nutrition education programme on the metabolic syndrome in type 2 diabetes mellitus patients at a level 5 Hospital in Kenya: “a randomized controlled trial”

**DOI:** 10.1186/s40795-020-00355-6

**Published:** 2020-08-04

**Authors:** Ann Watetu. Thuita, Beatrice Nyanchama Kiage, Arnold N. Onyango, Anselimo O. Makokha

**Affiliations:** School of Food and Nutrition Sciences, Department of Human Nutrition Sciences, Jomo Kenyatta University of Agriculture and Technology, Juja, Kenya

**Keywords:** Metabolic syndrome, Type 2 diabetes mellitus and cardiovascular risk

## Abstract

**Background:**

Type 2 diabetes mellitus (T2D), is a life-threatening condition of global public health concern. It worsens in the presence of the metabolic syndrome (MetS), a complex disorder characterized by co-occurrence of at least three of such factors as hypertension, obesity, dyslipidemia and insulin resistance. However, lifestyle interventions reduce the risk of both MetS and T2D, and nutrition education can empower individuals on the appropriate, lifestyle changes. The aim of the current study was to evaluate the effect of a nutrition education programme, with and without inclusion of peer to peer support, on MetS in T2D patients.

**Methods:**

This was a randomized controlled trial with two intervention groups and one control. One of the intervention groups involved a nutrition education programme with peer-to-peer support (NEP); the other involved only the education program, while the control received standard care. Each group had 51 participants. The nutrition education programme was conducted for 2 h per week for 8 weeks. In addition, the NEP had weekly peer-to-peer interactions for 8 weeks. All groups had follow-up sessions for 6 months. Data on MetS risk factors as well as food intake patterns and physical activity levels were taken at baseline and at different time points during the study. Analysis of Co-variance and regression were used in the analysis.

**Results:**

The MetS prevalence improved in the NEP (90 to 52%) and NE (86 to 69%), while it worsened in C (88 to 91%). There was improvement in the mean values of the anthropometric parameters in the NEP and NE which worsened in the control group. There was a general improvement in mean values of blood lipids, fasting blood glucose and HbA1c in all the groups, with NEP showing the greatest improvements, followed by NE, except for triglycerides and HDL where the control group had better improvement than the NE. Changes in the anthropometric and metabolic indicators mirrored the changes in food intake patterns and physical activity, where the greatest improvements occurred in the NEP.

**Conclusions:**

Nutrition education with inclusion of peer to peer support was of clinical benefit in improving metabolic outcomes and reducing MetS in T2DM patients.

**Trial registration:**

The study has been registered retrospectively by Pan African Clinical Trial Registry; Registration No: PACTR201910518676391.

## Background

Type 2 Diabetes mellitus is a metabolic disorder, characterized by poor glycemic control due to insulin insufficiency and insulin resistance [1]. It is a global public health problem whose prevalence is increasing worldwide and especially in developing countries [1–4]. It is aggravated in the presence of the metabolic syndrome (MetS); a cluster of interrelated clinical factors, that include insulin resistance, dyslipidemia, excess weight and elevated blood pressure [5–7].

Due to increased prevalence of obesity, surplus energy intake and sedentary lifestyle, Mets in Type 2 Diabetes mellitus patients is becoming a worldwide epidemic [8]. A high prevalence of between 50 and 80% of MetS in Type 2 Diabetes mellitus patients, using different definitions, has been reported in different parts of the world [9–14]. Similar high prevalence has been reported across the globe in the general population [11, 15–18] Presence of MetS in Type 2 diabetes mellitus patients leads to an increase in microvascular and macrovascular complications [5–7, 17–21].

Unhealthy lifestyle has been associated with faster progression of Type 2 diabetes mellitus as well as MetS in Type 2 diabetes mellitus patients [22–24]. However, this can be improved through lifestyle interventions such as improved nutrition and increased physical activity [22–25]. Unfortunately, achieving these lifestyle modifications is usually very challenging due to poor self-control, lack of information, financial constraints among others. For this reason, well designed health education advocacy and awareness creation programmes on positive lifestyle changes should be promoted [24, 26].

Peer to peer social and emotional support has been shown to help people apply disease management or prevention plans in daily life, and links individuals with clinical, community, and other resources [27–29]. Additionally, studies have shown that the effectiveness of diabetes education on lifestyle modification can be enhanced through inclusion of peer to peer support [28, 30–34]. However, despite the established role of lifestyle intervention and peer to peer support in improving Type 2 diabetes mellitus and MetS, its contribution to Type 2 diabetes mellitus and MetS management in Africa, including Kenya, is not well established. Moreover, data on the existence of MetS in Type 2 Diabetes mellitus population, as well as, intervention to address MetS in Type 2 diabetes mellitus in in Kenya have not been reported. Therefore, the purpose of the present study, was to implement a nutrition education (NE) programme with peer to peer support, and evaluate its effect on the MetS and MetS risk factors in adults with Type 2 Diabetes mellitus.

## Methodology

### Study setting

The study was conducted at Thika Level 5 Hospital (TL5H) in Kiambu County, Kenya at the Diabetes Comprehensive Care Centre (DCC). The clinic attends to approximately one hundred patients per week. The DCC is an out-patient clinic that operates on a daily basis. Diabetic patients from Kiambu County and nearby areas attend the clinic on appointment days for routine monitoring of blood glucose, blood pressure and nutrition status (body mass index; BMI), as well as for treatment and collection of medication. Newly diagnosed patients with either Type 1 or Type 2 Diabetes mellitus are also referred here from neighboring health facilities for further management. The clinic serves both male and female patients with Type 1 and Type 2 diabetes mellitus. The patients are mainly from low and middle income backgrounds.

### Study design and ethics

This was a randomized controlled trial, with two intervention groups (nutrition education; NE and Nutrition education with peer to peer support; NEP) and a control group (C). The study was approved by the Kenyatta National Hospital-University of Nairobi Ethics and Research Committee (KNH-UoN-ERC), Permit No: KNH-ERC/A /232, and, the Kenya National Commission for Science, Technology and Innovation (NACOSTI); Permit No: NACOSTI /P/16/83452/10118. Study participants gave a written informed consent before the start of the study.

### Study participants

Study participants were men and women, aged 20–79 years, with Type 2 diabetes mellitus attending care at the Diabetes Comprehensive Care Centre (DCC) at TL5H. They were recruited during their daily clinic attendance while waiting to see a health professional. Recruitment was done over a period of 2 months from August 2016 to October 2016. All patients who met the following criteria were selected: patients suffering from Type 2 diabetes mellitus aged between 20 and 79 years, regular attendance at the DCC; not planning to move from the study area during the study period; not pregnant; with no complications such as renal failure, congestive heart failure, or stroke. A total sample size of 153 patients was recruited for the study.

### Sample size determination

To confer 90% power at 5% level of significance, and to detect an absolute effect size of 30% improvement on metabolic syndrome (MetS) in Type 2 diabetes mellitus patients (i.e. a decline from 90 to 65% Mets prevalence with intervention), we needed to include 46 study participants in each study arm using the formula by Armitage et al.*,* [35] and Lwanga and Lemeshow [36]. The sample size was subjected to a correction factor of 10% to cater for attrition, thus each arm had 51 participants making a total sample size of 153 patients.

### Randomization

**Fig. 1:**
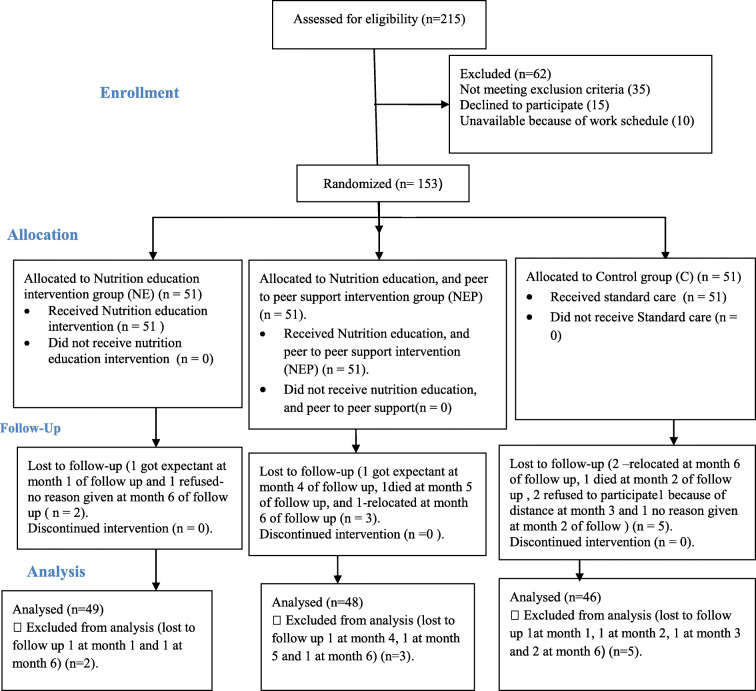
Flow of the participants throughout the study

The study consisted of two intervention groups and a control group. The Nutrition Education (NE) group received nutrition education; the Nutrition Education with Peer-to Peer support (NEP) group received nutrition education with peer to peer support; while the control group (C) received standard care. Participants were randomized to either NE or NEP or C groups by use of random numbers as shown in Fig. 1. To allow equal chances for participants, randomization was stratified on the basis of sex and age. Sealed sequentially numbered opaque envelopes per each stratum (1–3), mixed using the lottery method were used. The participants were requested to pick an envelope each and join their groups (1–3). A volunteer from each group was then requested to move forward and pick another envelop each, that contained their treatment allocation (NE, NEP and C). Upon confirmation of the treatment allocation, the participants were allocated to their treatment group by the principal investigator (PI), and the group members recorded. Each group was assigned 51 participants. After randomization baseline data was collected from all the participants. Randomization and flow of the participant throughout the study is as shown in Fig. 1.

### Intervention

Before random assignment to control or intervention groups, all study participants received standard education that covered content on diabetes pathophysiology, risk factors, symptoms, complications, hyperglycemia and hypoglycemia symptoms and foot care treatment goals and modalities. This was done by the principal investigator (PI) together with a clinician who runs the clinic (Registered Clinical Officer with a Bachelor of Science degree in Clinical medicine). The Standard Education relied on pictorial flip charts and additional learning material with diabetes management information. These were adapted from the diabetes prevention and management guidelines from the Ministry of Public Health and Sanitation (MoPHS), Kenya [37];, the NorvoNodisk Changing Diabetes poster, as well as diabetes posters from the Ministry of Health (MOH), Kenya, with supplementary information provided by the PI obtained by a review of different literature. Different teaching methods including lectures, discussions, demonstrations, role plays and group work were used to deliver the information. The participants also received standard care that included blood glucose and blood pressure monitoring, treatment for those with problem as well as education on diabetes care by a clinician on monthly basis.

After the standard education, the intervention groups (NE and NEP) underwent a nutrition education programme for 8 weeks, which also covered the importance of physical activity (NE group). The curriculum for this programme is provided in the Appendix. In addition, the NEP group was trained on peer-to-peer support. The nutrition education given to the NE and NEP intervention groups included weekly (120 min each) nutrition classes conducted over 8 weeks by the PI. The nutrition education curriculum was developed by the PI after review of related literature on nutrition management of Type 2 diabetes mellitus. The PI also applied her experience gained from practice as a nutritionist. The NE curriculum was written in English and supplemented by photos and illustrations to help the patient understanding the content better. It focused on nutrition in relation to diabetes; food portion control for weight reduction; healthier food choices; individualized meal planning,; glycemic index and glycemic loads of different food and their importance in blood glucose control; the food pyramid, and its use together with food exchange list in meal planning. Patients learnt about the basics food groups, the difference between simple and complex carbohydrates and their relation to glycemic index and glycemic load, fibre content of different cereals and starches, the difference between saturated and unsaturated fats and their relation to diabetes management; sources of protein and the different nutrient content of each, hidden calories contained in beverages, and the micronutrient and fiber values of fruits and vegetables. The nutrition education content was presented using lectures, demonstrations, discussions, and other participatory methods. The nutrition education curriculum was first tested in a subgroup (10% of sample) of patients not involved in the study before the actual implementation. The physical activity lesson was given to the intervention groups (NE and NEP group) in the last week of the education programme. The aim of the physical activity was to ensure that patients accumulate a minimum of 150 min of moderate intensity exercise each week from personal activity at home that includes walking, digging, jogging, cycling, house hold duty, aerobics and sport activities. The participants were encouraged to perform the exercise at least 3 days each week with no more than two consecutive days without exercise. During the physical activity lesson, the patients were led through the importance of physical activity in management of Type 2 diabetes. Additionally, demonstrations on activities they can do at home were done by a physiotherapist experienced in diabetes management t. The participants were encouraged to continue with the exercises at home in addition to normal routine work.

Participants in the NEP group were divided into small support groups (5–10 participants); depending on the location they came from as well as their age. After each education session, members of the support groups were encouraged to set and share with one another other weekly goals for specific changes in their eating and physical activity behavior. The goals were aimed at making healthy food choices, reduction of portion sizes and being active. The participants reported on their progress to the group members at the beginning of the next session. After the 8 week training, participants were followed monthly, and they presented their progress and new goals to the group members, for a period of 6 months. A trained peer educator living with diabetes for 13 years from Kenya Defeat Diabetes Association (KDDA) joined the PI during the monthly meetings and encouraged the participants in the peer support groups by sharing his experiences. Together with the PI he also assisted them review and adjust their goals during monthly meetings. Also, group counseling was done on each visit for participants requiring more support.

### Follow up

The follow up was done monthly after the intervention period. After the end of the 8 weeks intervention the patient were requested to be coming to the hospital monthly on selected days for follow up. At the start of the study the patient were given appointment cards developed by the PI indicating the day they were supposed to come for the appointment. The PI also got phone numbers for the participants which assisted in follow up. A call was given to the participant reminding them on the appointment day 1 week to the appointment day and 2 days to the appointment day to ensure they avail themselves. Those who did not turn up would be given another day and be reminded again of their appointment. For those who could not make to come after second reminder, they were followed in their home and requested to come for the appointment. This prevented loss to follow up. Patient in the NEP group continued with peer to peer support during the follow up period.

### Measurements

Measurements were taken on anthropometry and clinical data, blood pressure, blood glucose and lipid profile, as well as physical activity levels and food intake. A physician and clinical officer were also present during the study period to manage any patient requiring medical treatment.

### Anthropometry and clinical data

Anthropometric measurement that includes weight, height, waist and hip were collected using standard protocols [38, 39] at baseline, during monthly follow up and post evaluation after 6 months. Height and weight were measured using standard methods with the participants wearing light clothes and no shoes [38]. The weight was determined to the nearest 0.1 kg using a calibrated electronic weigh scale (Seca) and height to the nearest 0.1 cm using a stadiometer attached to the weighing scale. Body mass index (BMI) was then be calculated as weight (kilograms)/height (meters) 2 and classified as per WHO classification [38]. The waist circumference and hip circumference were measured according to standard guideline [39]. Waist circumference was measured mid-way between the lower rib margin and the iliac crest with flexible anthropometric tape to the nearest 0.5 cm while hip circumference was measured as the maximal circumference around the buttocks posteriorly and pubic symphysis anteriorly.

### Blood pressure

Blood pressure of the patient was also taken monthly. It was measured in the supine position using, a mercury sphygmomanometer (model: Autortensio® noSPG440) by trained nurses with at least a 10-min rest period before the measurement.

### Laboratory assay

Blood samples were collected from each participant while in a seated position after fasting for 8-12 h for determination of serum triglycerides (TG), total cholesterol (TC), high density lipoprotein (HDL-c), low-density lipoprotein cholesterol (LDL-c), glycated hymoglobin (HbA1c) at baseline and 6 month post intervention. Fasting blood glucose was determined monthly. Levels of serum triglycerides (TG), total cholesterol (TC), high density lipoprotein (HDL-c), low-density lipoprotein cholesterol (LDL-c), were determined by enzymatic method [40–46]. Glycated Hemoglobin (HbA1c) and blood glucose were determined using high-performance liquid chromatography and glucose oxidase method respectively [47, 48].

### Metabolic syndrome definition

Metabolic syndrome in the study was defined according to the definition of WHO [47] and “Circulation for Harmonizing the Metabolic Syndrome” criteria [2, 21]. The latter requires the presence of at least three of the following five components: Fasting blood sugar of 100 mg/dl or 5.6 mmol/l or drug treatment of elevated glucose, central obesity for Africans (waist circumference ≥ 94 cm in males and ≥ 80 cm in females), elevated triglycerides (≥1.7 mmol/l or 150 mg/dl and/or the use of triglyceride-lowering drugs), reduced HDL cholesterol (< 1.0 mmo/l or < 40 mg/dl in males and < 1.3 mmol/l or 50 mg/dl in females) and elevated blood pressure (systolic blood pressure ≥ 130 mmHg and/or diastolic blood pressure ≥ 85 mmHg and/or the use of antihypertensive drugs).

World Health Organization criteria also requires the presence of Type 2 diabetes mellitus, impaired glucose tolerance or insulin resistance, and any two of the following:(1) body mass index (BMI) ≥ 30 kg/m2 and/or waist-to-hip ratio > 0.90 (male), > 0.85 (female); (2) blood pressure ≥ 140/≥90 mmHg or on hypertension medication; and (3) triglyceride ≥1.7 mmol/Land/or HDL-C < 0.91 mmol/L (male), < 1.01 mmol/L (female).

### Physical activity

Physical activity data was collected using a physical activity questionnaire. It included questions asking the participants the type of activities they did, the time spent on each activity and number of days per week on each activity. The metabolic equivalent for each physical activity was tabulated and recorded. This was done at baseline, month 1, month 3 and month 6 post intervention.

### Dietary intake

This was collected by asking the participants 12 questions on healthy dietary choices adapted from perceived dietary adherence questionnaire (PDAQ) [49], dietary approach to stop hypertension questionnaire (DASH) [50] and medical nutrition therapy (MNT) [51, 52]. These questions sought to inquire whether the participants followed their commendation of; health diet plan, diet rich in fruits and vegetables, complex carbohydrates high in fibre, low glycemic index food that included whole grains, reduced intake of saturated fat and overall fat, included fish or fish products in their meal, reduced intake of sugars and sugar sweetened products, spaced carbohydrate intake, reduced intake of salt, included low fat food in the meal as well as, uptake of monosataurated and polysaturaed fat. The responses to the questions were based on a 7-likert scale.

### Data analysis

The data was analyzed using statistical package for social science (SPSS version 20). Data are present as means ± SD or SE for continuous variables and percentages for categorical variables. Chi square test and multinomial regression was used to compare groups for categorical variables and Analysis of Co-variance (ANCOVA) was used to compare difference of means between groups. Statistical significance was considered for p value < 0.05.

## Results

### Participants

One hundred and fifty-three participants (153; 40.5% male and 59.5% female) were included in the study. As shown in Table 1, there was no significant difference in the baseline characteristics of the study participants. A total number of 143 (93.5%) participants completed the study and were used for final analysis. The losses to follow up included 3partcipants in NEP, 2 participants in NE group and 5 participants in the control group as indicated in the flow diagram. The mean age of the participants was 56 years; with 46.4% of the participant having a family history of diabetes; 77.8% having poor glycemic control (HbA1c > 7%) and 58.2% had lived with diabetes for 1–5 years prior the study [53]. The prevalence of MetS was 86.3 and 88.2% as per WHO and Harmonized criteria respectively at baseline.

**Table 1:** Baseline characteristics of the study participants

Parameter		NEP	NE	C	***P*** Value
		Mean ± SD or n (%)	Mean ± SD or n (%)	Mean ± SD or n (%)	
**Age in years**^**a**^		57.0 ± 10.88	55.0 ± 12.34	56.0 ± 11.97	0.76
**YLWD**^**a**^		6.0 ± 7.10	7.0 ± 6.93	7.0 ± 6.63	0.63
**Gender**^**b**^	**Male**	17(33.3)	24(47.1)	21(41.2)	0.37
	**Female**	34(66.7)	27(52.9)	30(58.8)	
**Marital**^**b**^**status**	**Married**	45(88.2)	43(84.3)	41(80.4)	0.53
	**Divorced/separated/windowed**	6(11.8)	8(15.7)	10(19.6)	
**Income**^**b**^	**< 1000**	26 (51.0	21 (41.2)	25 (49.0)	0.17
	**1001–5000**	13 (25.5)	7 (13.7)	12(23.5)	
	**5001–10,000**	5 (9.8)	9 (17.6)	9 (17.6)	
	**> 10,000**	7 (13.7)	14 (27.5)	5(9.8)	
**Occupation**^**b**^	**Formal employment**	2(3.9)	1(2.0)	3(5.9)	0.75
	**Casual employment**	1(2.0)	4(7.8)	5(9.8)	
	**Farming**	22(43.1)	21(41.2)	20(39.2)	
	**Business**	15(29.4)	18(35.3)	15(29.4)	
	**Unemployed**	11(21.6)	7(13.7)	8(15.7)	
**Complication**^**b**^	**Foot disease**	5(9.8)	7(13.7)	5(9.8)	0.77
	**Eye problem**	13(25.5)	12(23.5)	11(21.6)	0.88
	**Kidney problem**	0(0)	2(3.9)	0(3.9)	0.11
	**Neuropathy**	1(2.0)	0(0)	3(5.9)	0.11
	**Arthritis**	6 (11.8)	7(13.7)	5(9.8)	0.83
**FHD**^**b**^	**Yes**	28 (54.9)	22(43.1)	21(41.2)	0.32
	**No**	23 (45.1)	29(56.9)	30(58.8)	
**Medicaltion**^**b**^	**Oral**	45 (29.4)	37 (25.2)	44(28.8)	0.08
	**Insulin**	9 (5.9)	6(3.9)	4(2.6)	0.32
	**Oral plus insulin**	2 (1.3)	3 (2.0)	3 (2.0)	0.88

^**a**^ data presented as mean ± SD

^**b**^Data presented as proportion (n) and percentages%

Statically significance at *p<*0.05; chi (^x^2) square test

n for all the groups (NEP, NE and C) =51

YLWD- years lived with diabetes

FHD – family history of diabetes

As shown in Table 2, there was no significant difference between the groups in the anthropometric (weight, BMI, WC, HC, WHR), clinical (SBP, DBP) and biochemical variables (HbA1c, TC, TG, HDL, LDL and FBS) at baseline. Furthermore as shown in Table 2, NEP group showed greatest significant reduction in weight (− 6.27 (0.87) kg; *p <* 0.01), BMI (− 2.37 kg/m (0.34); *p <* 0.01), WC (− 14.51(1.34) cm; *p <* 0.01, HC (− 6.16 (1.28) cm; *p <* 0.01) and WHR (− 0.027(0.008); *p* = 0.01) 6month post intervention, (Table 2). Moreover, Bonferroni post hoc comparison between groups showed that there was a significant difference (*p <* 0.01) between NEP and C in weight lost (6.89 kg), BMI (2.26 kg/m^2^) reduction, WC reduction (16.45 cm) and HC reduction (10.20 cm) 6 months post intervention. Additionally, significant difference (*P <* 0.01) was also seen between NEP and NE in weight lost (4.99 kg), BMI reduction (1.89 Kg/m^2^) and WC reduction (9.73 cm) as well as between NE and C in WC reduction (6.72 cm) and HC reduction (9.24) (Table 2).

**Table 2:** Changes in metabolic outcomes and differences between groups six-month post intervention

Parameter	Baseline data			Changes in clinical parameters six-month post intervention++					Differences between groups post intervention		
	NEP (***n =*** 51) Mean ± SD	NE (***n =*** 51) Mean ± SD	C (***n =*** 51) Mean ± SD	***P*** value	NEP (***n =*** 48) Mean (SE)	NE(***n =*** 49) Mean (SE)	C(***n =*** 46) Mean (SE)	***P*** value	NEP-NE	NEP-C	NE-C
**Weight**	72.06 ± 14.42	69.61 ± 10.22	71.91 ± 12.09	0.52	−6.27(0.87)	−1.27(0.84)	+ 0.63(0.87)	0.000	4.99**	6.89**	1.89
**BMI (Kg/m2)**	27.64 ± 5.72	26.34 ± 4.16	27.11 ± 4.04	0.38	− 2.37(0.34)	− 0.48(0.33)-	+ 0.29(0.34)	0.000	1.89**	2.26**	0.77
**WC (cm)**	101.92 ± 9.51	98.90 ± 9.71	101.71 ± 10.20	0.23	−14.51(1.34)	−4.78(1.29)4	+ 1.944(1.35)	0.000	9.73**	16.45**	6.72**
**HC (cm)**	106.16 ± 7.14	102.69 ± 11.90	106.17 ± 7.74	0.09	−6.16(1.28)	−5.2(1.24)	+ 4.04(1.29)	0.000	0.96	10.20**	9.24**
**SBP (mmHg)**	145.33 ± 21.33	146.04 ± 19.50	139.98 ± 18.66	0.25	−13.39(3.53)	−14.77(3.430	−5.30(3.56)	0.14	−1.38	8.09	9.47
**DBP (mmHg)**	87.88 ± 10.37	90.69 ± 8.79	88.12 ± 9.15	0.26	−1.58(198)	−5.17(1.92)	+ 2.41(1.99)	0.03	−3.58	3.99	7.57*
**HbA1C (%)**	8.81 ± 1.94	8.37 ± 1.81	8.28 ± 1.81	0.31	−2.04(0.39)	−1.48(0.39)	−0.73(0.40)	0.09	0.56	1.30*	0.75
**FBG (mmol/l)**	11.12 ± 2.73	11.41 ± 4.40	10.50 ± 2.77	0.38	−2.59(0.66)	−2.95(0.64)	−1.55(0.68)	0.31	−0.36	1.04	1.40
**TC (mmol/l)**	5.23 ± 1.43	4.77 ± 1.07	4.91 ± 1.13	0.12	−0.38(.24)	+ 0.13(0.23)	+ 0.30(0.24)	0.12	0.51	0.69*	0.17
**TG (mmol/l)**	2.32 ± 1.37	2.00 ± 0.92	2.39 ± 0.89	0.16	−0.67(0.18)	− 0.15(0.18)	− 0.58(0.18)	0.10	0.52	0.09	−0.43
**HDL (mmol/l)**	1.30 ± 0.29	1.55 ± 0.39	1.31 ± 0.31	0.07	+ 0.34(0.073)	+ 0.06(0.071)	+ 0.31(0.074)	0.01	−0.28*	− 0.03	0.25*
**LDL (mmol/l)**	2.45 ± 1.48	2.37 ± 1.21	2.05 ± 1.14	0.24	+ 0.38(0.24)	+ 0.53(0.23)	+ 1.23(0.24)	0.04	0.15	0.86*	0.71*
**WHR**	0.96 ± 0.07	0.98 ± 0.08	0.95 ± 0.09	0.23	−0.027(0.008)	+ 0.002(0.007)	+ 0.008(0.008)	0.01	0.30*	0.36*	0.01

Data are presented as mean ± standard deviation or SE of the mean. ANCOVA was used for between-groups comparisons, with a significance level of P* < 0.05 and p** < 0.01

*BMI* body mass index, *WC* waist circumference, *HC* hip circumference, *WHR* waist-to-hip ratio, *SBP* systolic blood pressure, DBP: diastolic blood pressure, *FBG* fasting blood glucose, *HDL* high density lipoprotein, *LDL* low density lipoprotein, *TG* triglycerides, *TC* total cholesterol and HbA1c –glycated hymoglobin, *NEP* Nutrition education peer to peer support group, *NE* Nutrition education intervention group, *C* control group, *Kg* kilogram/metre2, *Cm* centimeter, mmhg- Millimeters of mercury, mmol/l = milimmole per litre

Adjusted for age, gender, marital status, education level, family history of diabetes, years lived with diabetes, complications and medication use.

Significant increase in HDL + 0.34(0.07) mmol/l; *p* = 0.1 was also seen in the NEP group, 6 months post intervention. Furthermore, post hoc comparison between groups showed a significant difference between group in HDL levels; − 0.28 mmol/l between NEP and NE and + 0.25 mmol/l between NE and C. Moreover significant reduction in DBP -5.17(1.92) mmhg was also seen in the NE group six-month post intervention (Table 2). Post hoc comparison between group in DBP reduction showed a difference 7.57 mmhg between NE and C that was significant (*P <* 0.05). Additionally, post hoc comparison between groups showed a significant difference in TC levels (0.69 mmol/l, *p* < 0.05) between NEP and C as well as in HbA1c levels (1.30%) between NEP and C. Moreover, post hoc comparison between groups was also significant in LDL levels between NEP and C (0.86 mmol/l) as well as between NE and C (0. 71 mmol/l), Table 2. There was no significant mean difference for the other metabolic parameters between the intervention groups (NEP and NE) and C group (Table 2).

As shown in Table 3, there was no significant difference in MetS prevalence and metabolic risk factors (increased WC, high WHR, high FBS, elevated BP, elevated TG, reduced HDL, elevated TC, Elevated LDL) as well as in high BMI(> 25 kg/m^2^) between group at baseline. However, the NEP intervention group significantly reduced MetS (Odd Ratio; OR = 0.08, Confidence Interval; CI = 0.02–0.28, *P <* 0.01 and OR 0.20, CI = 0.06–0.68, *P <* 0.01) as defined by harmonized and WHO criteria respectively compared to control (C) group (Table 3). Additionally, comparison of NE and C, six-month post intervention, also showed a significant reduction in MetS^b^ prevalence (as defined by WHO) in the NE group (OR = 0.20, CI = 0.06–0.68, *P* = 0.01) (Table 3). Additionally comparison of NEP and C 6 month post intervention showed a significant reduction in prevalence of participants having increased WC(OR = 0.03, CI = 0.003–0.22, *P* = 0.001), increased WHR (Or = 0.09, CI = 0.01–0.93, *p* = 0.043), elevated BP as per harmonized and Who criteria respectively (OR = 4.17, CI = 1.59–10.91, *P <* 0.01 and OR = 4.29, CI = 1.67–11.03, *P <* 0.01), increased TG (OR = 0.3, CI = 0.13–0.75, *P* = 0.01) as well as reduced HDL (OR = 17.55, CI = 2.05–150.37,*p <* 0.01) respectively (Table 3).. Similarly comparison of NE and C 6 month post intervention showed a significant reduction in elevated BP as per harmonized criteria (OR = 0.40, CI = 0.16–0.97, *P* = 0.04) as well as WC (OR = 0.09, CI = 0.01–0.07, *P =* 0.02) (Table 3). Moreover in comparison to C group, a significant increase was seen in participants having a BMI of 18.5–24.9 kg/m2 6 month post intervention in the NEP group (OR = 4.62, CI = 1.32–16.20, *P =* 0.017) as well as in the NE group (OR = 4.25, CI = 1.09–16.59, *P =* 0.038) (Table 3). Furthermore compared to C group the NEP and NE group also showed a significant increase in participants having less than 3 MetS risk factors as per harmonized criteria definition(OR = 24.03, CI = 5.78–99.88, *P <* 0.01 and OR = 5.63, CI = 1.63–21.77, *P <* 0.01). Additionally, a reduction in prevalence of participants having dyslipidemia was also seen in NEP group six-month post intervention (OR = 0.30, CI = 0.13–0.7, *P <* 0.01) in comparison to control (Table 3).

**Table 3:** Prevalence of MetS risk factors at baseline and six-month post intervention

Parameter		Before the Intervention					Six-month Post intervention						
		NEP n (%)	NE n (%)	C n (%)	χ^**2**^	***P*** value	NEP n (%)	NE n (%)	C n (%)	NEP		NE	
										Odd ratio ^**a**^ (95% CI)	***P*** value	Odd ratio ^**b**^ (95% CI)	***P*** value
**High HbA1c**		43(84.3)	38(74.5)	38(74.5)	1.89	0.39	23(47.9)	24(49.0)	16(34.8)	2.08(0.85–5.09)	0.111	2.04(0.84–4.92	0.114
**High FBS**		51(100.0)	51(100)	51(100)			38(79.2)	41(83.7)	42(91.3)	2.91(0.82–10.36)	0.100	2.30(0.56–7.34)	0.114
**High WHR**		45(88.2)	48(94.1)	40(78.4)	5.64	0.06	42(87.5)	46(93.9)	45(97.5)	0.09(0.01–0.93)	0.043*	0.28(0.03–3.00)	0.294
**BMI**	**> 18.5–24.9**	18(35.3)	18(35.3)	17(33.3)	11.10	0.09	29(60.4)	19(38.8)	13(28.3)	4.62(1.32–16.20)	0.017*	4.25(1.09–16.59)	0.038*
	**> 25–29.9**	15(29.4)	27(52.7)	25(49.0)			13(27.1)	26(53.1)	22(47.8)	1.08(0.31–3.81)	0.915	3.13(0.85–11.51)	0.086
	**> 30–34.9**	23(45.3	9(10.8)	11(17.6))			8(12.5)	4(8.2)	13(23.9	Reference			
**Elevated WC**		47(92.2)	45(88.2)	47(92.2)	0.629	0.73	28(58.3)	42(85.7)	46(97.8)	.0.03(0.003–0.22)	0.001**	0.09(0.01–0.72)	0.024
**Elevated BP**^**a**^		37(72.5)	45(88.2)	37(72.5)	4.84	0.089	24(50.0)	24(49.0)	37(80.4)	4.17(1.59–10.91)	0.004**	4.29(1.67–11.03)	0.002**
**Elevated BP**^**b**^		34(66.7)	38(74.5)	28(54.9)	4.388	0.11	23(47.9)	21(42.9)	32(69.6)	0.395(0.16–0.97)	0.043*	0.33(0.14–0.78)	0.412
**Elevated TG**		32(62.7)	28(54.9)	39(76.5)	4.083	0.130	17(35.4)	31(63.3)	30(65.2)	0.31(0.13–0.75)	0.010 *	0.59(0.37–2.10)	0.785
**Reduced HDL-C**^**a**^		19(37.3)	11(21.6)	14(27.5)	3.126	0.21	1(2.1)	5(10.2)	10(21.7)	17.55(2.05–150.37)	0.009**	2.66(0.80–8.53)	0.111
**Reduced HDL-C**^**b**^		8(5.7)	5(9.8)	5(9.8)	1.333	0.567	1(2.1)	1(2.0)	1(2.2)	0.59(0.03–11.32)	0.730	0.91(0.05–16.86)	0.949
**Dyslipidemia**		35(68.6)	32)(62.7)	39(76.5)	1.700	0.32	18(36.7)	31(61.3)	30(65.2)	0.30(0.13–0.74)	0.008	0.89(0.37–2.11)	0.788
**Elevated TC**		26(51.0)	16(31.4)	22(43.1)	4.083	0.13	15(31.2)	18(36.7)	23(50.0)	2.45(0.99—6.04)	0.051	0.96(0.41–2.25)	0.918
**LDL**		29(59.9)	15 (29.4)	24(47.1)	7.994	0.244	25(52.1)	35(71.4)	31(67.4)	1.96(0.805–4.75)	0.14	0.87(0.35–2.16)	0.77
**MetS**^**a**^		46(90.2)	44(86.3)	45(88.2)	0.378	0.828	25(52.1)	34(69.4)	42(91.3)	0.82(0.02–0.28)	0.000**	0.20(0.06–0.68)	0.01*
**MetS**^**b**^		46(90.2)	45(88.2)	41(80.2)	2.318	0.31	28(58.3)	38(77.6)	41(89.1)	0.20(0.067–0.57)	0.003**	0.50(0.17–1.52)	0.22
**MetS risk factors**^**a**^	**1–2**	4(7.8)	7(13.8))	6(11.8)	13.323	0.101	20(41.7)	11(22.4))	5(10.8)	24.03(5.78–99.88)	0.000 **	5.63(1.63–21.77)	0.007**
	**3**	10(19.6)	21(41.2)	13(25.5)			20(41.2)	27(55.1)	28(60.8	3.32(11.10–99.60)	0.033*	1.48(0.59–3.74)	0.404
	**4–5**	37(62.7)	23(45.1)	32(63.3)			8(16.7)	12(26.4)	20(43.5)	reference			
**MetS risk factors**^**b**^	**1–2**	5(9.8)	6(11.8)	10(19.6)	2.492	0.65	20(41.7)	11(22.4)	5(10.8)	10.37(2.72–39.53)	0.001**	2.75(0.79–9.57)	0.011*
	**3**	25(49.0)	26(51.0)	22(43.1)			20(41.2)	27(55.1)	28(60.8)	2.92(0.94–9.10)	0.65	1.73(0.67–4.45)	0.258
	**4–5**	21(41.2)	19(37.3)	19(37.3)			8(16.7)	11(22.4)	13(28.3)	reference			

Data are presented as proportion; n (percentages; %) chi-square (χ2) test; *statistical significance at *p* value< 0.05.

BMI obese > 30 kg/m2, Elevated Waist hip ratio (WHR) > 0.90 for men and > 0.85 for women, Elevated blood pressure ^a^ > 140/90 mmHg or treatment of previously diagnosed hypertension (WHO criteria); Elevated blood pressure ^b^ > 130/85 mmHg or treatment of previously diagnosed hypertension (harmonized criteria), Reduced serum HDL cholesterol (a) < 0.9 mmol/L for men or < 1.0 mmol/L for women or specific treatment for this abnormality (WHO criteria); Reduced serum HDL cholesterol ^b^ < 1.0 mmol/L for men or < 1.3 mmol/L for women or specific treatment for this abnormality (harmonized criteria), Elevated triglycerides (TAG) > 1.7 mmol/L or specific treatment for this abnormality (both criteria), Waist circumference (WC) ≥94 cm for men or ≥ 80 cm for women, Elevated TC > 5.2 mmol/l, Elevated LDL-cholesterol> 2.6 mmol/l, Dyslipidemia- reduced HDL(< 0.9 mmol/L for men or < 1.0 mmol/L for women or specific treatment for this abnormality) or /and elevated TG(> 1.7 mmol/l) MetS^a^: Harmonized criteria; MetS^b^: WHO criteria, NEP; Nutrition education peer to peer support group, NE; Nutrition education group, C; control group,χ2**;** chi square Odd ratio^a^ –comparison MetS parameters of NEP and C, Odds ratio^b^-comparison of MetS parameters of NE and C, CI; confidence interval.

Adjusted for age, gender, education level, marital status, years; lived with diabetes, family history of diabetes, and complications.

As shown in Table 4, there was no significant difference between the groups in the mean frequency of consumption of different types of food at baseline. High means > 4 days per week of inclusion of high fat food, sugar/ sweetened beverages and refined carbohydrates, were seen in all participant at baseline. However, there was great change in fat consumption pattern by all the groups at month 3 and 6 month post intervention, where the mean for high fat food consumption dropped to 1 day per week or less. A significant improvement (*p <* 0.01) was seen in the NEP group 3 month and 6 month post intervention in inclusion of vegetables (5.84 ± 1.89 & 6.02 ± 1.59, *p <* 0.01), spacing carbohydrates (5.86 ± 1.90&5.29 ± 1.45, *p* < 0.01) and limiting sodium (5.10 ± 1.81 &5.54 ± 1.37; *p <* 0.01) in their meals, Additionally, an improvement was also seen in the NEP group in terms of including high fibre food for > 5 days a week in the meal (5.85 ± 0.99, *p <* 0.01) 6 month post intervention. Moreover participants in the NEP group also included low fat food in their diet for > 4 days a week (4.29 ± 2.08, *p <* 0.01) 6 month post intervention and carbohydrates of low glycemic index for > 3 day per week (3.94 ± 1.49, *p <* 0.01 and 3.85 ± 1.46, *p <* 0.01) 3 and 6 month post intervention respectively (Table 4).

**Table 4:** Frequency of food consumption for the participant at baseline, month 3 and month 6 post intervention

Variables	Baseline				Month 3				Diffrences between group between group at month 3			NEP (***n*** = 48) Mean ± SD	Month 6	C (***n =*** 46) Mean ± SD	***P*** value	Differences between group at month 6		
	NEP (=51) Mean ± SD	NE (***n*** = 51) Mean ± SD	C (***n*** = 51 Mean ± SD	***P*** value	NEP (***n*** = 51) Mean ± SD	NE (***n*** = 50) Mean ± SD	C (***n*** = 49) Mean ± SD	***P*** value					NE (***n*** = 49) Mean ± SD					
																NEP-NE	NEP-C	NE-C
1	2.61 ± 1.26	2.12 ± 0.99	2.22 ± 0.73	0.12	4.31 ± 1.33	3.20 ± 1.74	1.92 ± 1.64	< 0.01	1.09(0.32)**	2.36(0.32)**	1.26(0.32)**	5.15 ± 1.50	4.00 ± 1.80	2.71 ± 1.06	0.01	1.14(0.32)**	2.38(0.32)**	1.24(0.32)**
2	2.96 ± 1.23	2.55 ± 1.46	2.78 ± 1.38	0.34	3.58 ± 2.31	2.68 ± 2.17	2.27 ± 2.44	0.02	0.83(0.47)	1.26(0.48)*	0.43(0.47)	3.98 ± 2.08	2.88 ± 2.03	2.74 ± 2.10	0.02	1.03(0.43)	1.21(0.44)*	0.18(0.43)
3	3.02 ± 0.68	2.80 ± 0.66	2.80 ± 0.63	0.17	5.84 ± 1.89	3.88 ± 2.45	3.63 ± 2.62	< 0.01	1.76(0.46)**	2.01(0.46)**	0.26(0.46)	6.02 ± 1.59	4.06 ± 2.12	4.06 ± 2.12	0.000	1.83(0.41)**	1.92(0.42)**	0.9(0.41)
4	2.49 ± 0.90	2.52 ± 1.13	2.51 ± 1.13	0.99	3.94 ± 1.49	2.60 ± 1.51	1.77 ± 1.61	< 0.01	1.36(0.32)**	2.17(0.31)**	0.81(0.31)*	3.85 ± 1.46	2.61 ± 1.53	1.73 ± 1.51	0.000	1.24(0.31)**	2.1(0.32)**	0.87(0.31)*
5	2.68 ± 1.49	2.84 ± 1.16	2.52 ± 1.29	0.53	3.50 ± 1.35	2.46 ± 1.50	1.43 ± 1.27	< 0.01	1.01(0.28)**	2.02(0.28)**	1.02(0.28)**	5.85 ± 0.99	4.14 ± 1.47	2.67 ± 1.23	0.000	1.76(0.26)**	3.22(0.26)**	1.46(0.26)**
6	4.80 ± 0.80	4.22 ± 1.05	4.52 ± 1.11	0.06	0.69 ± 1.16	1.02 ± 1.13	1.22 ± 1.49	0.11	−0.36(0.27)	−0.56(0.26)	− 0.20(0.26)	0.58 ± 0.87	1.04 ± 1.14	1.30 ± 1.49	0.071	−0.46(0.24)	− 0.72(0.25)	− 0.26(0.24)
7	0.71 ± 1.22	1.00 ± 1.32	0.92 ± 1.29	0.38	0.71 ± 1.22	1.02 ± 1.33	0.94 ± 1.31	0.35	−0.47(0.26)	−0.38(0.26)	0.09(0.25)	0.67 ± 1.22	1.02 ± 1.35	0.93 ± 1.34	0.16	−0.42(0.27)	−0.34(0.27)	0.09(0.27)
8	5.84 ± 0.46	5.78 ± 0.51	5.82 ± .059	0.30	0.71 ± 1.64	0.70 ± 1.43	1.29 ± 1.72	0.12	−0.067(0.30)	−0.65(0.31)	− 0.59(0.30)	0.58 ± 1.30	0.69 ± 1.44	1.30 ± 1.76	0.19	−0.07(0.31)	−0.69(0.32)	− 0.60(0.31)
9	2.80 ± 0.89	2.86 ± 0.89	2.70 ± 1.04	0.68	5.86 ± 1.90	3.88 ± 2.45	3.63 ± 2.62	< 0.01	1.76(0.46)**	2.01(0.46)**	0.26(0.46)	5.29 ± 1.45	3.78 ± 1.43	2.28 ± 1.28	0.00	1.58(0.29)**	3.10(0.29)**	1.52(0.29)**
10	2.31 ± 1.49	2.33 ± 1.61	2.76 ± 1.42	0.24	3.82 ± 2.59	1.64 ± 2.07	0.93 ± 1.75	< 0.01	2.35(0.44)**	3.05(0.44)**	0.70(0.43)	4.29 ± 2.08	2.38 ± 1.86	1.96 ± 1.81	0.00	1.95(0.40)**	2.38(0.40)**	0.43(0.40)
11	2.98 ± 1.01	3.01 ± 0.88	2.98 ± 0.84	0.95	5.10 ± 1.81	3.76 ± 2.55	2.00 ± 2.02	< 0.01	1.124(0.44)*	2.88(0.44)**	1.74(0.43)**	5.54 ± 1.37	4.16 ± 2.24	2.21 ± 1.95	0.00	1.35(0.39)	3.24(0.40)**	1.89(0.39)**
12	1.90 ± 1.36	1.86 ± 1.31	1.82 ± 1.48	0.96	1.84 ± 1.33	1.86 ± 1.32	1.63 ± 1.47	0.66	0.05(0.28)	0.28(0.28)	0.23(0.28)	4.85 ± 1.20	4.93 ± 1.17	5.06 ± 1.34	0.87	−0.10(.26)	−0.24(0.26)	0.14(0.26)

Data presented as Mean ± sd; statistically significant = *p <* 0.05; ** significant at *p <* 0.01; *significant at *p <* 0.05; NEP-nutrition education peer to peer support, NE- nutrition education group and C – control group.

Variables definition

1. On how many days per week in the last 1 month did you follow a healthful eating plan
2. On how many days per week in the last 1 month did you did you eat three to five or more servings of fruits each day
3. On how many days per week in the last month did you eat three to five or more servings of vegetables each day
4. On how many days per week in the last month did you include high fibre such as whole grain, legumes in your diet
5. On how many days per week in the last month did you include low caloric of low glycemic index food in your meal
6. On how many days per week in the last 1 month did you include high fat foods like fatty meat, skin on chicken, highly fried foods
7. On how many days per week in the last month did you include fish in your meal each day
8. On how many days per week in the last month did you include sugar and sweetened beverages
9. On how many day per week in the last month did you space your carbohydrates throughout the day
10. On how many days per week in the last month did you include low sodium diet in your meal
11. On how many days per week in the last month did you include low fat foods like skimmed milk, lean meat, lentils
12. On how many days per week in the last one moth did you prepare your food with unsaturated fats like canola oil, olive oil, sunflower oil

As shown in Table 5 the participant in the all the groups had an average of 1000 MET minute physical activity levels at baseline. The physical activity level improved significantly (*p <* 0.01) in the NEP group at month 1, 3 and 6 respectively after the intervention (+ 570.92; 174.51 MET minutes, + 919.21; 192.96MET minutes and + 1105.36; 220.60) MET minute compared to the other groups (Table 5). Comparison of changes in physical activity levels between the groups showed significant difference between NEP and C at month 3 and month 6 post intervention. However no significant difference was found between NEP and NE as well as NE and C in physical activity level changes 1, 3 and 6 months post intervention (Table 5).

**Table 5:** Physical activity levels of the participants at Baseline, Month 1, Month 3 and Month 6 post intervention

	NEP Mean (SE)	NE Mean (SE)	C Mean (SE)	P value	Difference in Change of physical activity between groups		
					NEP-NE Mean (SE)	NEP-C Mean (SE)	NE-C Mean (SE)
**Baseline**	1024.32 (139.38)	1049.70 (138.231)	1015.39 (137.82)	0.955	−25.38 (197.96)	8.94 (197.10)	34.32 (194.66)
**Changes in PA at Month 1** **Changes in PA at Month 3** **Changes in PA at Month 6**	+ 570.92 (174.51) + 919.21 (192.96) + 1105.36 (220.60)	+ 116.21 (113.08) + 256.92 (193.45) + 380.12 (216.86)	+ 2.28 (172.56) + 15.71 (197.02) + 103.40 (223.92)	0.056 0.004 0.006	454.71 (247.87) 662.29 (275.29) 725.24 (311.00)	568.63 (246.79) 903.50 (277.27)* 1001.96 (316.12)*	113.93 (243.73) 241.22 (275.48) 276.72 (311.37)

Data presented as Mean (SE).

Physical activity levels presented as MET minutes per week.

NEP: Nutrition education peer to peer support group; NE: Nutrition education group and C: control group.

SE: Standard error of the mean.

PA- physical activity level.

MET; Metabolic equivalent.

*-statistically significant at *p <* 0.01

Adjusted for age, gender, marital status, education level family history of diabetes and years lived with diabetes.

## Discussion

The current study determined the effect of a nutrition education programme with or without on peer to peer support on metabolic syndrome and metabolic risk factors in type 2 diabetes patients. The 8-week nutrition education programme (Curriculum attached in the appendix) equipped participants with more detailed knowledge on diabetes-related nutrition and importance of physical activity than the standard education such patients usually receive in diabetes clinics in Kenya. The control group in the present study received the standard education. In addition to the standard education, one of the two intervention groups (NE) received the detailed nutrition education programme, and the other received the detailed nutrition education programme beefed up with a peer-to-peer support component (NEP group).

While there was worsening in mean values of most of the anthropometric and metabolic parameters such as weight, BMI, DBP, and LDL in the control group during the 6 months of the study, most of these parameters improved significantly in the NE and NEP group, with the NEP group achieving greater improvement than the NE group (Table 2). However, there were improvements in both HbA1c and fasting blood glucose in all the groups, and the means for these parameters were not statistically different at 6 months (Table 2). This may be attributed to the anti-diabetes medications taken by all the groups, which lowered the blood glucose.

Similar results were obtained for the prevalence of the metabolic syndrome and its risk factors (Table 3), where there were improvements in HbA1c and FBG in all the groups; worsening in the anthropometric risk factors and BP in control group; and improvements in the latter for the NE and NEP groups. Elevated TC and LDL worsened in the control group but improved in the NE and NEP groups. Prevalence of elevated TG dropped significantly in the NEP group, but increased in the NE and Control groups. There was reduction in prevalence of the rest of the blood lipid profile components in all groups, with greater improvements in NEP and NE than C.

Improvements in blood lipid profiles even in the control group may be due to the effects of antidiabetic medicines, such as metformin which has been shown to not only improve blood glucose but also blood lipids [54].

Overall, there was a worsening in the prevalence of the metabolic syndrome and its risk factors in the control group, and an improvement of the same in the NE and NEP groups, with greater improvements in the latter. The better improvements in Mets in the NEP than in NE and in the latter than in the C group may be attributed to different degrees of improvement in the food intake patterns and physical activity levels attained (Tables 4 and 5).

Nutrition education is a main component in diabetes education and has been shown to improve dietary behavior and clinical outcomes in persons with diabetes [26, 30, 55]. Previous studies have demonstrated that nutrition education or, lifestyle interventions aimed at correcting dietary behavior and enhancing physical activity in management of Type 2 Diabetes and MetS have a positive outcome in metabolic parameters [24, 26]. Inclusion of peer to peer support in the lifestyle intervention have been shown to have a better clinical outcome [31, 56, 57]. The results of the current study are in agreement with these previous studies.

A previous study showed strong correlation between BMI and WC with glycaemia, triglyceride, HDL and blood pressure [58] with reduced level of BMI and WC being associated with low MetS. In the current study, the NEP group that had the strongest reductions in BMI also had the strongest reduction in the prevalence of TG, but the NE group had a greater drop in the prevalence of BP. The results for BP might be confounded by the effects of anti-BP medication.

As found in the current study, nutrition education and other health education intended to improve dietary habits and physical activity have been previously shown to improve dietary behavior, physical activity and clinical outcomes in persons with Type 2 diabetes Mellitus [26, 30, 55].

In interpreting the results of this study, some limitations need to be considered. The study period was limited to 6 months and this allowed assessment of short-term effects of the intervention. Longer periods of follow-up have been recommended in order to understand more of the sustainability of a peer-led intervention program and also in order to ensure long-term reduction of MetS risk factors. Additionally, the study was carried out in a public hospital set-up where patient population is of middle and low income hence the results can only be compared to a similar population. On the other hand, the high retention rate (93.7%) and the positive feedback obtained from the participants during the monthly follow-up was in was a strength of the study. The current study was also unique as it combined a comprehensive nutrition education programme with peer to peer support in the management of Type 2 Diabetes.

The current study reported significant improvement of metabolic parameters and MetS prevalence on application of lifestyle intervention and might be a useful base for community based study targeting Type 2 Diabetes population.

## Conclusion

The detailed nutrition education programme offered to type 2 diabetes patients in this study significantly improved the MetS and its risk factors in type 2 diabetes patients. Moreover, combining the nutrition education programme with peer-to-peer support resulted in significantly greater benefits in reduction of the Mets in type 2 diabetes. Therefore, such a programme can be recommended for inclusion in diabetes management programmes for improved health outcomes. Nevertheless, future studies should focus on improving the training contents and longer-term monitoring to achieve greater improvements.

## Data Availability

All the data set used and/or analyzed during the current study are included in this manuscript, Pan Africa Clinical trial Registry and in an attached supplementary data file.

## Abbreviations

MetS: Metabolic syndrome
HbA1c: Glycated hymoglobin
WHO: World Health Organization
WC: Waist circumference
HDL-c: High Density Lipoprotein cholesterol
TG: Triglyceride
TC: Total Cholesterol
LDL-C: Low Density Lipoprotein
BMI: Body Mass Index
WHR: Waist Hip Ratio
CVD: Cardiovascular Disease
TL5H: Thika Level 5 Hospital
DCC: Diabetes Comprehensive Care Centre
NE: Nutrition Education Group
NEP: Nutrition Education Peer to peer support Group
MET: Metabolic Equivalent
GPO/POD: Glycerol Phosphate Oxidase Peroxidase
CHOD/POD: Cholesterol Oxidase Peroxidase
KNH-UoN/ERC: Kenyatta National Hospital-University of Nairobi Ethical Research Committee
NACOSTI: National Commission for Science Technology and Innovation
ADDRF: Africa Doctoral Dissertation Research Fellowship
APHRC: Africa Population and Health Research Center
IDRC: International Development Research Centre
SD: Standard Deviation
SPSS: Statistical Package for Social Sciences
